# Precise Spatiotemporal Control of Optogenetic Activation Using an Acousto-Optic Device

**DOI:** 10.1371/journal.pone.0028468

**Published:** 2011-12-09

**Authors:** Kaiyu Wang, Yafeng Liu, Yiding Li, Yanmeng Guo, Peipei Song, Xiaohui Zhang, Shaoqun Zeng, Zuoren Wang

**Affiliations:** 1 Institute of Neuroscience, State Key Laboratory of Neuroscience, Shanghai Institutes for Biological Sciences, Chinese Academy of Sciences, Shanghai, China; 2 Graduate School of Chinese Academy of Sciences, Shanghai, China; 3 Britton Chance Center for Biomedical Photonics, Wuhan National Laboratory for Optoelectronics, Huazhong University of Science and Technology, Wuhan, China; 4 Key Laboratory of Biomedical Photonics of Ministry of Education, Huazhong University of Science and Technology, Wuhan, China; Harvard University, United States of America

## Abstract

Light activation and inactivation of neurons by optogenetic techniques has emerged as an important tool for studying neural circuit function. To achieve a high resolution, new methods are being developed to selectively manipulate the activity of individual neurons. Here, we report that the combination of an acousto-optic device (AOD) and single-photon laser was used to achieve rapid and precise spatiotemporal control of light stimulation at multiple points in a neural circuit with millisecond time resolution. The performance of this system in activating ChIEF expressed on HEK 293 cells as well as cultured neurons was first evaluated, and the laser stimulation patterns were optimized. Next, the spatiotemporally selective manipulation of multiple neurons was achieved in a precise manner. Finally, we demonstrated the versatility of this high-resolution method in dissecting neural circuits both in the mouse cortical slice and the *Drosophila* brain *in vivo*. Taken together, our results show that the combination of AOD-assisted laser stimulation and optogenetic tools provides a flexible solution for manipulating neuronal activity at high efficiency and with high temporal precision.

## Introduction

Experimental manipulation of neural activity in a spatially- and temporally-specific manner facilitates the study of neural circuit function [1]. Beyond traditional electrophysiological methods, rapidly developing optogenetic tools now allow non-invasive manipulation of the activity of selected neuronal populations expressing light-activatable ion channels or pumps, which induce membrane depolarization and hyperpolarization, respectively [2], [3]. The blue light-activatable channelrhodopsin-2 (ChR2) and its ameliorated versions have emerged as the most efficient proteins for optogenetic activation of neurons [4]–[6]. In most applications, blue light is delivered to the ChR2-expressing neurons via microscope objectives [7], optical fibers [8], or a light emitting diode (LED) [9]. Despite the high efficiency of neuronal activation by these methods, selective excitation of single neuron is difficult to achieve, partly due to the light dispersion and scattering and partly because ChR2-expressing neurons are often adjacently localized, making it difficult to achieve specificity.

To realize the selective excitation of particular neurons of interest, one solution is to use genetic means to label specific neurons [10] (e.g. using *CaMKII* promoter to label glutamatergic neurons and using *parvalbumin* promoter to label parvalbumin interneuron [11]. In addition, illumination of individual neurons in a spatially restrictive manner could be an alternative solution. Compared with wide-field light sources, collimated and focused laser light has much restrictive spatial distribution, making it suitable for the illumination of a small area. However, illumination of ChR2-expressing neurons with focused laser light usually failed to excite neurons reliably, largely due to the small inward current caused by the limited recruitment of ChR2 proteins using restrictive laser light [12]. To overcome this problem, two main kinds of approaches have been developed. In one, the spatial distribution of laser activation was expanded to cover a large area of the cell membrane, enabling parallel illumination of multiple sites to synchronically activate enough ChR2 proteins and thus evoke a large current [13], [14]. In another approach, by taking advantage of the long open time of ChR2 proteins [15], a restrictive laser spot could be sequentially moved across the whole cell to excite ChR2 proteins in a saturating manner [12]. However, the limited scanning speed and fixed scanning route of galvanometer-driven scanning mirrors weakened the ability of the sequential laser stimulation in manipulating the activity of multiple neurons simultaneously, which is usually necessary in studies of neural circuits [16]. As compared with reported two-photon laser stimulation approaches [12]–[14], random activation of ChR2 proteins by quickly moving the laser spot through a combination of acoustic-optic deflector (AOD) and single-photon laser requires less laser energy, thus reducing putative photo- and heat-damage.

AOD is a device which could change the pathway of laser beam in an ultra-fast (>100 sites per ms) and inertial-free manner [17], [18]. In addition, by changing the amplitude of the sound waves, the intensity of laser beam could be modulated, thus enabling very flexible control of the laser stimulation [18]–[20]. Combined with an ultraviolet (UV) laser, AOD has been successfully used to uncage glutamate and efficiently activate neurons at multiple points [21], [22], although the identities of neurons activated by uncaged glutamate are uncertain. To obtain a better optically control of specific, identifiable neurons at multiple points with high spatiotemporal resolution, we used the AOD-assisted blue laser stimulation to manipulate the activity of neurons genetically labeled with channelrhodopsins, including either ChR2 or the ameliorated chimera variant of channelrhodopsin ChIEF [5]. The performance of this system in activating ChIEF was first examined in HEK 293 cells and cultured neurons. By varying the moving speed and moving path of the laser, we optimized the stimulation pattern. Then, by distributing the laser stimulation on multiple neurons, a precisely spatiotemporal manipulation of activities of multiple neurons was achieved. Finally, the usefulness of this method in dissecting neural circuits was further investigated both in mouse cortical slice and *Drosophila* brain, demonstrating that the combination of AOD-assisted laser stimulation and genetically expressed channelrhodopsins is a valuable tool to study neural circuits.

## Results

**Figure 1:**
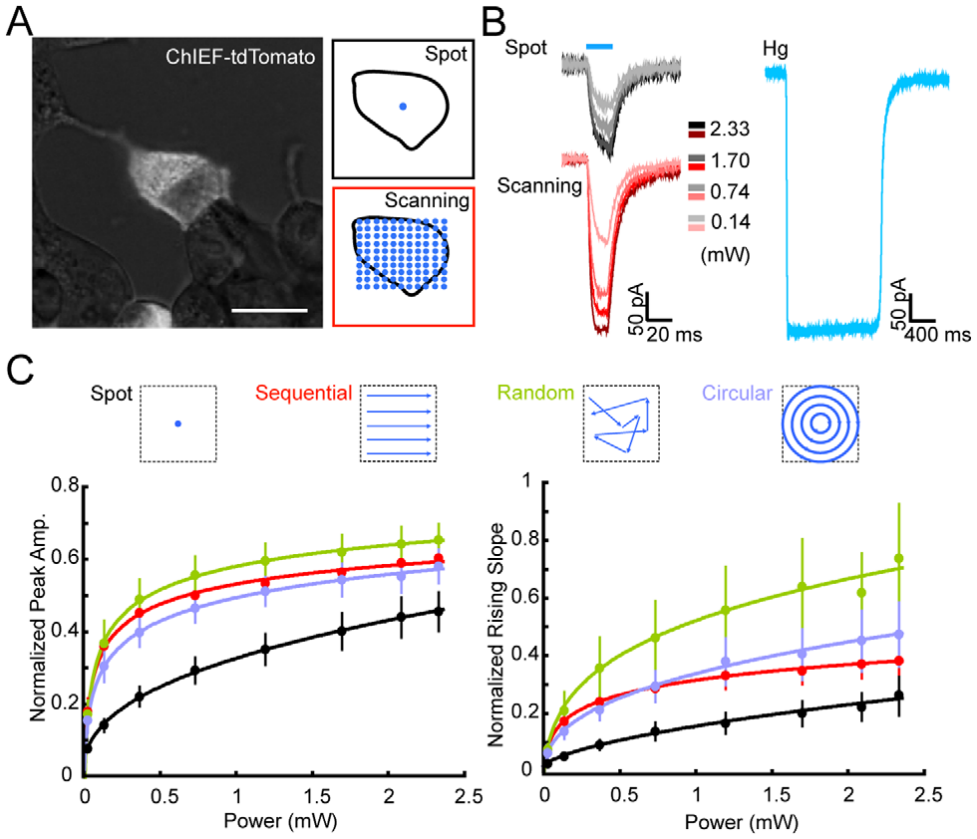
Efficacy of different modes of laser stimulation in activating ChIEF in HEK 293 cells. (A) Image of a HEK 293 cell expressing ChIEF-tdTomato under mercury lamp illumination. Boxes on the right depict the pattern of fixed-spot and whole-soma scanning stimulation. Scale bar, 20 µm. (B) Traces of whole-cell currents recorded from a ChIEF-expressing cell in response to fixed-spot (dark) and whole-soma scanning (red) stimulations at different levels of laser power, together with current evoked by mercury lamp illumination (cyan). (C) Dependence of the amplitude and the initial slope of the light-evoked currents on the laser power. The four stimulation patterns are illustrated above. Data points represent the mean ± SEM (n = 12), and curves represent the best logarithmic fit.

To activate channelrhodopsins, we used a blue laser (∼473 nm) in our system (Figure S1). After passing through two crosswise-orientated AODs, the laser beam was introduced into the optical path of a microscope and was focused by the objective to illuminate the sample. To evaluate the efficacy of the focused laser spot in depolarizing cells, ChIEF was expressed in HEK 293 cells, and light-evoked currents were measured by whole-cell recording (Fig. 1*A*). By focusing the laser spot on a fixed position on the cell, an inward current could be evoked when the laser intensity was above a threshold of ∼1 µW. As the laser intensity increased, the evoked current became larger, with a relationship well-fitted by a logarithmic function (Fig. 1*B–C*). However, as compared with those evoked by prolonged wide-field illumination with a mercury arc lamp, the amplitude of the laser-evoked current was smaller (Fig. 1*B–C*). This was presumably due to the limited area of ChIEF activation by a fixed laser spot (size ∼1.54 µm^2^). Since the activated ChIEF protein remains open for ∼10 ms (ref. [5], [23], rapidly shifting the laser spot to different locations on the recorded cell should in theory recruit more activatable ChIEF proteins, resulting in a larger current [12]. To examine this, we defined a pattern of illumination sites covering the whole cell and scanned the laser across the cell in an ultra-fast manner (10–50 µs/site) (Fig. 1*C*). This fast scanning stimulation resulted in enhanced light-evoked responses in HEK293 cells, with increases in amplitude and in the initial slope of the evoked currents (Fig. 1*B–C*). We also varied the pattern of scanning and found that, under most conditions, random scanning of the laser spot evoked faster and larger current responses than scanning with sequential and circular patterns (Fig. 1*C*). We reasoned that this superior activation ability of random scanning might be due to the larger area or smaller overlap covered by the laser spot in a defined length of time, as compared to that covered by the sequential or circular scanning. This is because the distance between two temporally adjacent stimulation positions was larger in random scanning than that in sequential or circular scanning. Thus, random scanning is the optimal method for effective activation of ChIEF-expressing cells and was used in subsequent experiments.

**Figure 2:**
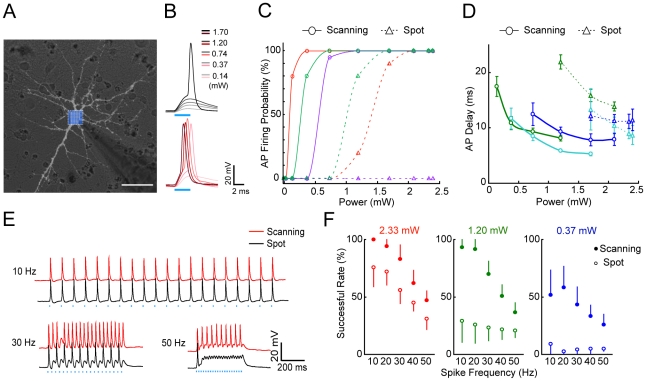
Characterization of neuronal activation by fixed-spot and random-scanning stimulations in cultured ChIEF-expressing hippocampal neurons. (A) Image of a recorded neuron under mercury lamp illumination. The box filled with blue dots indicates the area covered by random-scanning stimulation. Scale bar, 20 µm. (B) Membrane potential changes evoked by fixed-spot (gray) and random-scanning (red) stimulation using different levels of laser power (blue bar, duration of laser stimulation). (C, D) Probability and delay-on-onset of AP firing evoked by fixed-spot and random-scanning stimulation at different laser power levels for three example cells. Interpolation curves with the same color are for the same neuron. (E) Typical spike trains of different frequencies triggered by repetitive fixed-spot and random-scanning stimulation at a laser power of 2.33 mW (duration 6.2 ms). (F) Summary of the success rate of evoking APs, as defined by the percentage of successful AP initiation in 20 trials at different frequencies for three different levels of laser power (n = 7). Error bars, SEM.

To further characterize the performance of this AOD-assisted scanning method for neuronal excitation, ChIEF was expressed in low-density cultured hippocampal neurons and light-evoked currents were measured by whole-cell recording (Fig. 2*A*). Random scanning of the entire soma of ChIEF-expressing neurons was much more efficient than fixed-spot illumination of the soma in evoking action potentials (APs, Fig. 2*B*), and the reliability (firing probability measured in 20 trials at 5 Hz) of evoking APs increased with the laser power. Figures 2*C and 2D* depict the probability and the delay-of-onset of AP firing observed for scanning versus fixed-spot stimulation, respectively, in three example cells. Similar data were observed for 12 other cells. In addition, scanning stimulation was more reliable in triggering AP trains at different stimulation frequencies than fixed-spot stimulation (Fig. 2*E*). We attributed this reliability to the larger depolarization evoked by random scanning stimulation and the more rapid onset of AP firing, which shortened the time required for the next AP initiation. Moreover, APs could also be triggered by rapid scanning stimulation of multiple selected dendritic sites (Figure S2). One concern with light manipulation of neuronal activity is potential photodamage by the laser light [16]. In our system, the blue laser was focused on a spot (∼1.4 µm FWHM, full width at the half maximum) that covered only an area of 1.54 µm^2^ at the focal plane. For random scanning, most recorded neurons were reliably excited with a laser pulse of a power <∼1.2 mW and a duration <10 ms, for total energy load (<12 µJ) much lower than those associated with previously reported two-photon stimulation of ChR2-expressing neurons [12]–[14]. Indeed, after repeated laser stimulation of the same neuron over prolonged periods (>1 hour), no obvious change was found in the morphology or membrane properties of the stimulated neurons. Thus, AOD-assisted laser stimulation is an efficient and non-invasive method for activating ChR2-expressing neurons.

**Figure 3:**
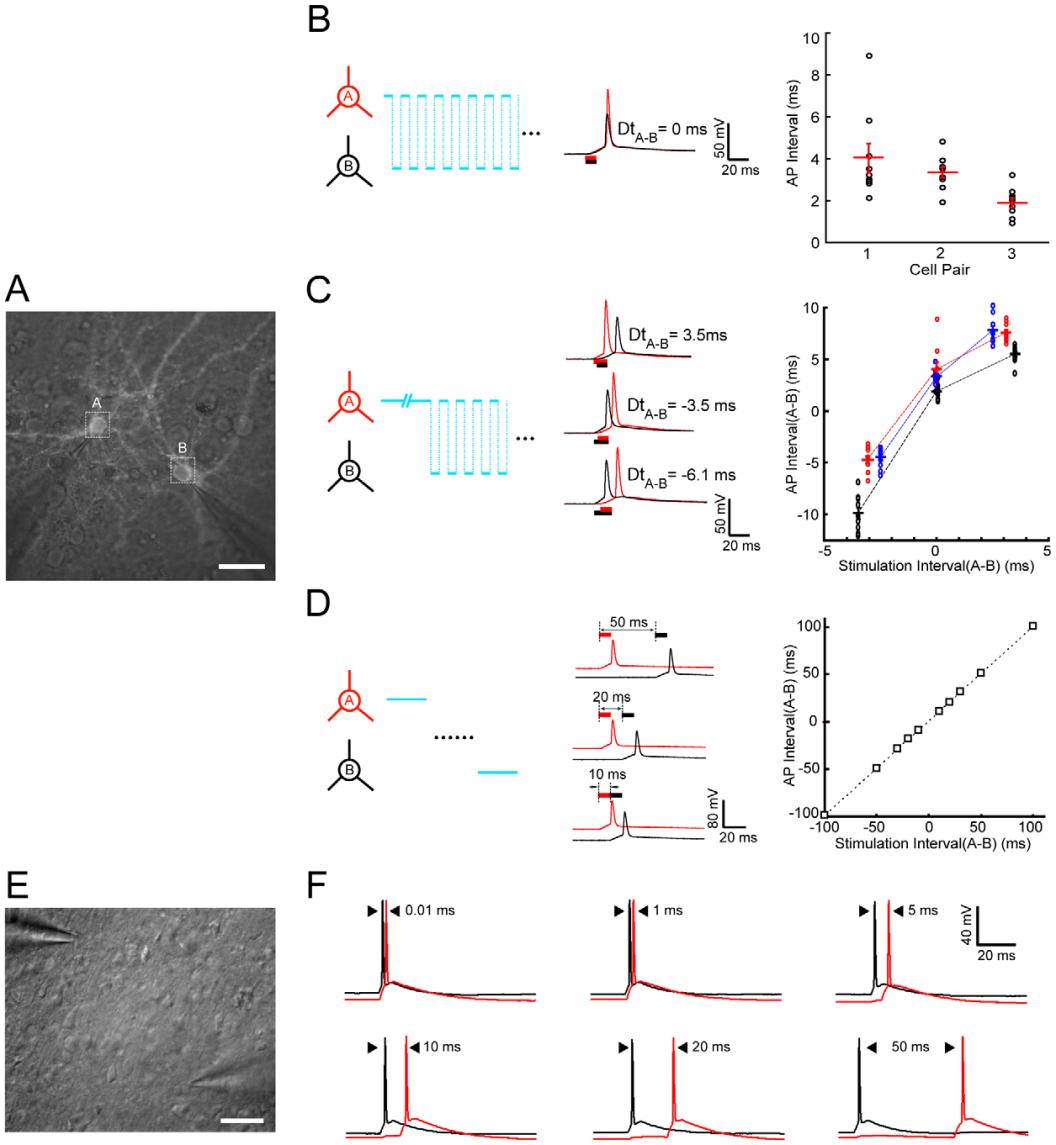
Temporal control of AP firing in two neurons. (A) Fluorescence image of two cultured ChIEF-expressing cortical neurons with dual whole-cell recording. Scale bar, 20 µm. (B) Synchronous activation of two cells using alternating scanning at random spots on the soma (depicted on the left). Traces are example recordings of coincident APs evoked by alternating stimulation of the two recorded cells. (C) Sequential activation of two cells with millisecond intervals by continuous random-scanning stimulation of the first cell for a defined duration, followed by alternating scanning stimulation of both cells (depicted on the left). Traces are example recordings of sequential AP firing in same cell pairs evoked by different stimulation patterns intended to induce specific intervals and spiking order. The plot on the right summarizes the results obtained from three recorded cell pairs and depicts the relationship between the intended and observed spiking intervals in two cells. Negative intervals represent spiking of the opposite order. (D) Sequential activation of two cells with intervals longer than 10 ms was achieved by complete whole-soma scanning stimulation of the first cell followed by stimulation of the second. Traces are example recordings of sequential AP firing in same cell pairs evoked by sequential whole-soma stimulation at different intervals. The plot summarizes results obtained from three recorded cell pairs and depicts the relationship between the intended and observed spiking intervals in two cells. (E) Bright-field image of two ChR2-expressing pyramidal cells in the S1 slice prepared from the AAV injected mouse (P60). Scale bar, 50 µm. (F) Similar to (D), Sequential AP firing of this PC pair was evoked at the inter-stimuli intervals of 0.5, 1, 5, 10, 20, and 50 ms respectively.

The ultra-fast scanning and patterned multi-site stimulation abilities of our system allow the possibility of manipulating the activity of multiple neurons in a temporally specific manner. To assess this, we carried out experiments on both dissociated neuron cultures and cortical slices. We first transfected high-density cultured cortical neurons with lentivirus encoding ChIEF-tdTomato and performed dual whole-cell recordings from two ChIEF-expressing neurons while applying laser scanning stimulation on two neurons with specific patterns (Fig. 3*A*). For temporal control of AP firing in the two neurons, three scanning approaches were used. First, for simultaneous firing, laser stimulation was alternately applied to two cells at random spots on the soma (Fig. 3*B*). This resulted in synchronous firing of two neurons expressing similar levels of ChIEF with a coincidence of <5 ms. Second, continuous random laser scanning was applied to the soma of one neuron for a chosen duration before scanning was alternated between the first neuron and the second neuron. This initiated AP firing of the first neuron before the second, with an interval determined by the chosen duration of the initial continuous scanning (Fig. 3*C*). Depending on the ChIEF expression level in the two cells, the initial period of continuous scanning varied. However, a roughly linear relationship between the intended and the observed intervals of sequential AP firing were obtained for three pairs of recorded neurons (Fig. 3*C*). Thirdly, for sequential activation of two neurons with intervals >10 ms, we triggered an AP with complete random soma scanning of the first neuron before moving the stimulation spot to trigger an AP in the second neuron (Fig. 3*D*). In this case, the intervals of evoked APs in the two cells closely followed those observed for the sequential light stimulation of two cells.

We also examined the precision of the AOD-assisted scanning method to temporally control spiking activity of multiple neurons in mouse neocortical slices. The expression of ChR2 in the mouse somatosensory cortex (S1) was achieved by stereotaxically injecting an Adeno-associated virus expressing NpHR3-EYFP-2A-ChR2-mCherry [24]. Strong expression of ChR2 in a limited number of cortical neurons around the injection site after 30 days could be observed in the brain slice. We then simultaneously made whole-cell recording of two ChR2-expressing pyramidal cells (PCs) to monitor their activities (Fig. 3*E*). Similar to that observed in the cultured neurons, we could readily control neuronal spiking in these two PCs in the brain slice with high temporal precision by steering the laser spot onto the two neurons with different time intervals starting from 0.01 ms (Fig. 3*F*). As compared to observations in cultured neurons, the higher efficacy of single-spot laser stimulation (with shorter pulse) for inducing spikes in the brain slice might be partially due to light scattering by the tissue surround and covering the surface area of the neuron. Indeed, fast-scanning stimulation did not evoke a much larger current than the fix-spot stimulation. Also, this higher efficacy might be partially due to robustly higher level of ChR2 expression in the slices through the *in vivo* viral transduction approach. Moreover, in the neocortical slice, the AOD-assisted laser spot stimulation was capable of reliably inducing high frequency spiking of fast-spiking (FS) GABAergic interneuron up to 200 Hz (Figure S3 *C1–C2*). Together, these results demonstrate that combination of the AOD-assisted laser stimulation system and the optogenetic tools offers great flexibility and precision in manipulating spikes and spike timing of multiple neurons in a neural circuit.

**Figure 4:**
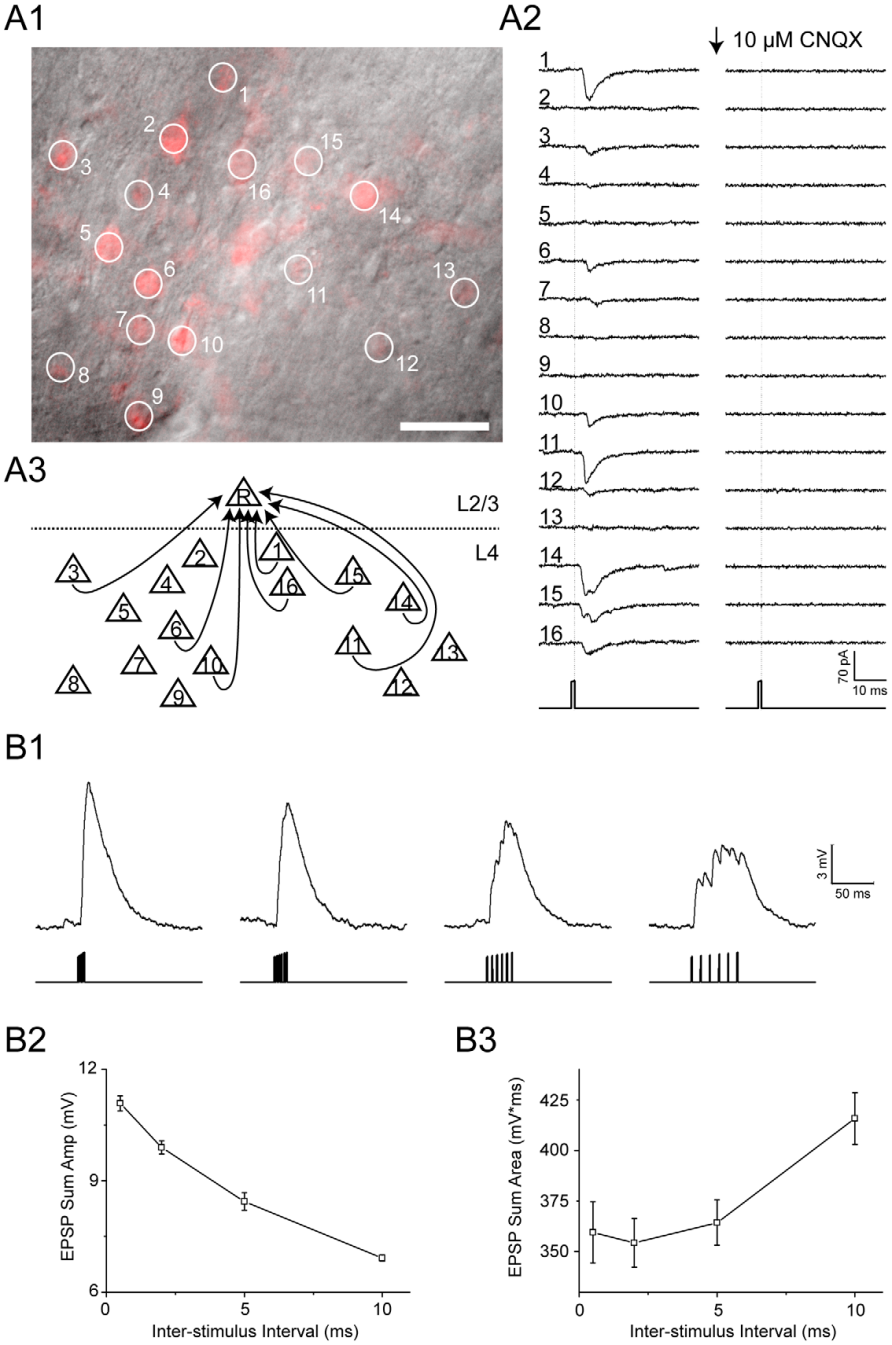
Application to synaptic connection mapping and temporal integration. (A1) a visual filed under 40× objective, showing the sparse distribution of ChR2-expressing neurons (in red) in layer 4 of the S1 slice prepared from AAV virus injected mice. Numbered circles refer to the laser activation sequence to these circled neurons. Scale bar, 50 µm. (A2) Postsynaptic current traces (averaged from 5 sweeps) recorded from a layer 2/3 ChR2-negative PC following the sequential activation of these 16 layer 4 ChR2-expressing neurons shown in A1 by AOD-assisted laser pulses (1 ms in duration, 0.75 mW). The AMPA receptor antagonist CNQX was applied in the bath solution at time indicated by the arrow. The dotted vertical lines indicate the off-timing of the laser pulse. (A3) A schematic diagram illustrates the monosynaptic connections from 8 layer 4 PCs to the recorded lay2/3 PC, based on the results shown A2. (B1) Traces represent the summed membrane potential change of the layer 2/3 neuron when cells #1, 3, 4, 14, 15 and 16, 6 layer 4 PCs illustrated in A3 are laser-activated to fire single spikes consecutively at 0.5, 2, 5, 10 ms intervals, respectively. The vertical lines indicate the times of sequential laser stimulation to these 6 layer 4 neurons. (B2, B3) The mean peak amplitudes (B2) and area magnitude of summed postsynaptic potentials from the experiments shown in B1. Error bars, SEM from 10 trials.

We further evaluated the applicability of this AOD-assisted light stimulation system for mapping functional synaptic connections in the mouse barrel cortex. We first demonstrated that the spiking probability of ChR2-expressing neurons was highly dependent on the laser power by applying AOD-assisted laser spot stimulation to their somas (Figure S3 *A1–A2*). The system could achieve specific induction of the firing of a single neuron as long as the neighboring ChR2-expressing neurons were more than 10 µm away (Figure S3 *B1–B3*). Such spatial resolution of the light excitation of single neurons in brain slices may endow the AOD-assisted system the applicability to map functional synaptic connectivity within a neural circuit. To directly demonstrate this, S1 cortical slices were made as above described, and the expression of ChR2 was observed in a cluster of layer 4 neurons in the cortical S1 area (Fig. 4*A1*). The synaptic connections from these ChR2-expressing layer 4 neurons to a layer 2/3 PC were mapped. After whole-cell recording on a ChR2-negative layer 2/3 PC was achieved, the AOD-assisted laser pulses (at 0.75 mW, 1 ms duration and 200 ms intervals) were steered to the soma of the 16 ChR2-postive layer 4 neurons to trigger spikes one-by-one (Fig. 4*A1*). We detected postsynaptic currents with varied amplitudes in layer 2/3 PC when 8 layer 4 neurons were excited by the AOD-assisted laser spot individually, suggesting cells #1, 3, 6, 10, 11, 14–16 formed synapses onto layer 2/3 PC with varying strengths (Fig. 4*A2–A3*). The mapped L4→L2/3 connections were all excitatory fast glutamate transmission because the postsynaptic currents were completely blocked by the application of AMPA subtype glutamate receptor antagonist CNQX (10 µM) in the bath solution (Fig. 4*A2*). We also noted that the onset delays of those evoked excitatory postsynaptic currents was fixed around 2.9±0.7 ms (n = 8), suggesting convergent monosynaptic inputs from these eight layer 4 PCs to the layer 2/3 PC. Moreover, since the AOD-assisted laser stimulation allows rapid transition of activation sites within 10 µs, we further examined the temporal summation of synaptic inputs in the layer 2/3 PC by choosing six strong L4→L2/3 connections from the above identified sub-circuit (Fig. 4*A3*). When these 6 synaptic connections were consecutively activated at time intervals ranging from 0.5 to 10 ms, we observed a gradual reduction of peak amplitude of the summed response but an increase of mean depolarization value (Fig. 4*B1–B3*). This result suggests a strong dependence of synaptic integration function on the input time intervals, which is consistent with previous study using a rapid uncaging system with an dual galvanometer-based scanning system [25].

**Figure 5:**
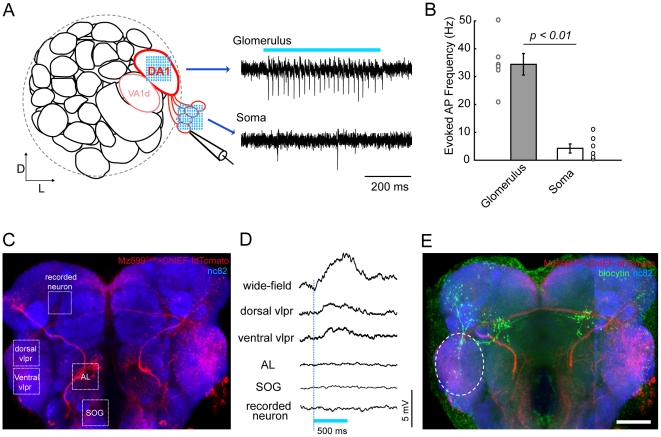
Specific activation of ChIEF-expressing neurons in transgenic *Drosophila* brain. (**A**) Schematic illustration of one antennal lobe in the *Drosophila* brain (left). Three glomeruli (DA1 and VA1d are dash circled, with DC3 beneath them) are labeled in the Mz19-Gal4 line, and the projection neurons (PNs) of the DA1 glomerulus are shown in red. Loose-patch recordings were made on DA1 PNs while laser scanning stimulation was provided either on the glomerulus or on the soma. Spike trains could be reliably evoked when stimulating the glomerulus but not when stimulating the soma (right). (**B**) Laser stimulation on the glomerulus is more capable of activating PNs than stimulation on the soma (n = 6, error bars: SEM). (**C**) Fluorescent image of a brain of a *Mz699-Gal4>UAS-ChIEF-tdTomato* transgenic fly. Cells expressing ChIEF are shown in red, and the whole brain was counterstained with the synaptic marker mAb nc82 to visualize brain structures (blue). Different stimulation sites are indicated by the white dashed boxes. (**D**) Membrane potential changes were measured by whole-cell recording while light stimulation was provided (Blue bar, duration of the laser stimulation). Significant depolarizations could be evoked by wild-field blue light illumination or laser stimulation on the ventrolateral protocerebrum (VLPR), but not on the antennal lobe (AL), the sub-esophageal ganglia (SOG), or the recorded neuron. (**E**) Post-hoc staining of the recorded neuron (green) showed the morphological overlap between the recorded neuron and the candidate upstream ChIEF-expressing neurons (white dashed ellipse). Scale bar, 20 µm.

The applicability of this method for studying neural circuits in the intact brain was also examined using *Drosophila* brains that specifically express ChIEF in defined neurons. As the fly brain is relatively small and much more transparent than the opaque mouse cortex, the focused laser light exhibit less scattering, and fix-spot stimulation could not evoke large current in ChIEF-expressing neurons. Thus, random-scanning stimulation was again used to reliably evoke AP trains in ChIEF-expressing neurons (Fig. 5*A–B*). Unlike mammalian neurons, APs were more likely to be evoked by light illumination of the neurites, but not the soma, of ChIEF-expressing neurons. This could be explained in part by the idea that the AP initiation site in *Drosophila* neurons may be located at sites away from the soma [26]. As observed in the cortical slice, by specifically activating different sets of ChIEF-expressing neurons, the inputs from these neurons could be mapped to other neurons in the brain (Fig. 5*C–E*). The above results suggest the usefulness of this AOD-assisted method for rapid mapping of functional connections in the intact brain. Furthermore, the capability of activating multiple neurons with defined spatiotemporal patterns also allows for studying synaptic integration in neurons.

## Discussion

### Using AOD-assisted system to study neural circuits

With the improvement of techniques available for studying nervous system, our understanding of how neural circuits behave is becoming more thorough. It is now obvious that merely exciting or shutting a vast of neurons en masse is not sufficient to elucidate how the brain works, as suggested by the complexity of neuronal components belonging to specific circuits, and the various ways in which these components orchestrate to endow the circuits with different functions [27]. Traditional optogenetic methods generally activate or inhibit many neurons within a region in a coarse manner [27]. Despite the advances obtained with these methods, it is still difficult to exactly understand the function of specific circuits or individual neurons. To achieve a fine manipulation of a neural circuit optogenetically, the ability to precisely control the behavior of individual neurons or neuron groups is required. The combination of AOD-assisted laser stimulation with genetically expressed channelrhodopsins is very suitable for these tasks. Unlike galvanometer-driven mirror system which steers the laser spot by mechanic movement of the mirror, AOD is inertia-free and enables ultra-fast and flexible beam steering. In addition, the motion path of the laser spot could vary as the deflection ratio of the virtue grating can be readjusted at every time point. Thus, the laser spot can move between any two locations in the visual field with an extremely high refreshing rate (in our system is ∼100,000 sites per second), which enables the activation of multiple isolated neurons selectively, an ability difficult to achieve with the galvanometer-driven mirror system.

One concern in using laser to evoke APs in individual neurons is that the channelrhodopsins expression level and intrinsic properties vary between neurons, so the delay of AP firing after laser stimulation might be different, which would be a hurdle to overcome when synchronized firing or sequential firing of neurons with desired intervals is required [28]. As it would be difficult to change channelrhodopsins level or neuronal properties, modulation of the laser stimulation on each neuron becomes the best alternative. The AODs can adjust not only the laser stimulation time allotted to each neuron, but also the laser intensity at any site, which offer a very flexible way to control the AP firing time in every neuron (Fig. 3).

### Comparison with other methods

AODs have long been used in manipulating neuronal activity. Combined with UV laser and caged glutamate, it is convenient to activate multiple neurons in an ultra-fast way [22], [29]. However, there are two main concerns when using this combination of methods in dissecting neural circuits. First, due to the abundant distribution of glutamate receptors in different cells and the complicate neuronal composition at any site in the neural circuit, restrictive uncaging of glutamate usually cannot achieve selective activation of an individual neuron [30]. Second, as the uncaged glutamate might not be degraded or removed efficiently at sites outside synapses, repeated laser stimulation could result in the accumulation of glutamate and thus alter the activity of the whole neural circuits [31]. To circumvent these problems, optogenetic tools turn out to be a promising alternative [31]. As it can be expressed genetically, only specific neurons could be manipulated, leaving all the other cells un-perturbed [32]. In addition, many optogenetic tools have high kinetics and keep a close pace with the stimulation light, relieving potential after-effects.

To obtain arbitrary spatial patterns of light stimulation, liquid crystal display (LCD) [33]–[35] and digital micromirror devices (DMD) [36]–[39] have been successfully applied to activate channelrhodopsins expressed in transgenic *C. elegans* and zebrafish with a high spatial selectivity [40]. Also, the recently developed methods which sculptured the shape of two-photon laser, by using the diffraction grating or the spatial light modulator (SLM), allowed manipulation of neurons located deep in the cortical slice [13], [14]. These three above-mentioned systems distribute the light to all the pixels and controlled the light intensity at each pixel in a parallel manner, leading to simultaneous stimulation of multiple sites. Meanwhile, the high refreshing rate of these parallel systems allow for fast altering of the stimulation patterns. However, as the light beam has to be expanded to illuminate all the pixels, a high power light source is indispensable to distribute enough power to every pixel.

Different from the above parallel illuminating methods which distributed the light to all pixels, the AOD system steers a single laser beam to different locations sequentially, so a laser of relative lower power would be enough to activate channelrhodopsins (Fig. 1). Also, as mentioned above, AOD is capable of changing the laser intensity at each spatiotemporal point, thus any stimulation site is independent from the other sites. As AODs are serial devices, simultaneous illumination of every point is difficult to achieve. However, taking advantage of the long open time of channelrhodopsins, neuronal activities of multiple neurons could be manipulated in a simultaneous manner (Fig. 3 and Fig. 4). Thus, the AOD system could be an alternative to the existing parallel illuminating methods. Furthermore, besides steering single-photon laser beam to manipulate channelrhodopsin-expressing neurons in samples which are relatively thin (e.g. cultured neurons), or in which the optogenetic tools are sparsely expressed, (e.g. the transgenic *C. elegans* or *Drosophila* brain), AOD-assisted two-photon laser microscopy with a high spatial resolution in the axial direction could also be used to selectively activate multiple sites within three-dimensional tissues [41].

### Further improvement of this system

In summary, we have established an AOD-assisted optogenetic method for manipulation of neuronal activities at multiple sites within neural circuits. In this work, we used an AOD system which provides laser beam scanning in two dimensions, where single-photon laser microscopy was sufficient to realize high-resolution light stimulation. Considering that the biological tissues are complex and three-dimensional, optical stimulation with a high spatial resolution both laterally and axially would be required. By using two sets of AODs, three-dimensional stimulation would be realized according to the three-dimensional functional imaging method [42]. Furthermore, the current system used a laser of a single wavelength (∼473 nm). Integration of a second laser of a different wavelength (e.g., yellow light for eNpHRs [43], [44] or Arch [45] ), together with an additional set of AODs, into this system will extend the capability for selective activation and inactivation of multiple neurons, providing a powerful tool for dissecting neural circuit underlying many brain functions.

## Methods

### Optical setup and laser stimulation

Our system was built as an independent integration based on an upright commercial microscope (FN1, Nikon, Japan). The main optical path was illustrated in Figure S1. In the laser stimulation system (QuickView-Stim, CBBMP, China), the laser beam from a blue light laser (MLL-III-473, λ = 473 nm, Changchun new industries optoelectronics tech, China) was coupled into a single mode fiber (SMF, NA = 0.11, OZ, Canada), and an aspheric lens (*f*_1_ = 4.5 mm) was equipped at the entrance of the SMF to enhance the coupling efficiency (>50%). An achromatic lens (*f*_2_ = 30 mm) was fixed right at the output end of the SMF to yield a collimating laser with beam diameter of 4.6 mm. Followed the achromatic lens, A half-wave plate was used to adjust the polarizing status of collimating laser. Before entering the microscope through a dual port (Y-QT, Nikon, Japan), the laser beam was sequentially passed through a pair of crosswise-oriented AODs (DTSXY-400-473, AA, France) and a scan lens. In the dual port, a dichroic mirror (DM505, Nikon, Japan) was used to reflect the 473-nm laser. After further passing through the objective (Nikon, NIR Apo 40×/0.80w), the laser beam was focused onto the focal plate to yield a restrictive laser spot (1.4 µm in diameter).

To accurately direct the laser spot to different locations on the sample, images of the sample were first captured by a CCD camera (IR-1000E, DAGE-MTI, USA) through an image grabber, and regions of interest (ROIs) were selected by software Image-Pro Plus (IPP, Media Cybernetics, USA) and the information of their positions was saved. An AOD-controlling program based on LabVIEW (National Instrument, USA) was developed, and termed as “Random PhotoStim” (RPS) program. The location information of the ROIs was transformed by RPS program into frequency signals to control the AODs, which determined the laser targeting site. At the same time, other parameters such as stimulation time, stimulation interval, and so on were configured. Then, stimulation according to the selected stimulation mode was started.

### DNA constructs and Transgenic fly

Mammalian codon-optimized full-length cDNA of ChIEF-tdTomato fusion protein [5] was cloned into pcDNA3.1 plasmid (Invitrogen, USA) for transfection of HEK 293 cells and low-density cultured hippocampal neurons, or a lentivirus based plasmid for lentivirus preparation, or pUAST plasmid for making transgenic fly. Transgenic fly was made following standard procedures, and transgenic lines with insertions of UAS-ChIEF-tdTomato on the second or third chromosome were obtained. Flies were reared in dark at 25°C on standard cornmeal agar medium plus all-trans-retinol. Adult males aged 1–3 days were used.

### Cell culture and *Drosophila* brain preparation

All experimental preparations followed the procedures approved by the Institute of Neuroscience, Chinese Academy of Sciences.

HEK 293 cells [46], [47] were plated at approximately 10^5^ cells per glass coverslip and maintained in DMEM with 10% fetal bovine serum and 0.2% penicillin-streptomycin. Cells were transfected with 2 µg of plasmids encoding ChIEF–tdTomato using calcium phosphate transfection. All recordings were performed 24–48 h after transfection, at room temperature (22–24°C).

Hippocampal neurons were prepared from postnatal day 0 (P0) Sprague-Dawley rat pups and plated on matrigel (BD Biosciences) coated glass coverslips (Assistant, Germany) at 35,000–50,000 neurons per cm^2^ in medium consisting of Neurobasal medium (Invitrogen), B-27 (Invitrogen) and Glutamax-I (Invitrogen). On the third day in vitro (DIV 3), when astrocytes have formed a monolayer over the entire coverslip, cells were treated with the mitotic inhibitor FUDR (5-fluoro-20-deoxyuridine, Sigma). Calcium phosphate transfection was performed at DIV 7 using 1 µg of plasmids encoding ChIEF-tdTomato per 12 mm coverslip. Recordings were performed 11–18d after transfection, at room temperature (22–24°C).

Dissociated embryonic mouse cortical neurons (E18) were plated on coverslips. Neurons were infected by the lentivirus encoding ChIEF-tdTomato at DIV 4. Recordings were performed 10–16 days after infection, at room temperature (22–24°C).

The *Drosophila* was anesthetized by cooling on ice for 30–45 sec. The brain was dissected out in extracellular solutions and the perineural sheath was removed with fine forceps. The dissected brain was placed with its anterior part upright on a dish covered with a thin sheet of Silgard, and immobilized with polyamides fibers fixed on a U-shape platinum bar.

### Electrophysiology

Whole-cell recordings were performed at room temperature with a Multiclamp 700B amplifier (Axon Instruments, USA) using low-resistance pipets (2–5 MΩ for HEK 293 cells, 3–7 MΩ for cultured neurons, and 9–12 MΩ for *Drosophila* neurons). The data was filtered at 2 kHz and acquired at 10 kHz. All reagents were purchased from Sigma-Aldrich unless otherwise indicated.

For whole-cell patch-clamp recordings of HEK 293 cells, the intracellular solutions contained (in mM) 130 K-gluconate, 10 EGTA, 1 MgCl_2_, 1 CaCl_2_, 10 HEPES, 2 MgATP, 0.3 Na_3_GTP (pH to 7.3). The extracellular solution contained (in mM) 150 NaCl, 5 KCl, 5 CaCl_2_, 1 MgCl_2_, 10 HEPES. (pH to 7.34). The cells were voltage-clamped at −40 mV.

For whole-cell patch-clamp recordings of cultured neurons, the intracellular solutions contained (in mM) 110 K-gluconate, 20 KCl, 5 MgCl_2_, 20 HEPES, 0.6 EGTA, 2 MgATP, 0.2 Na_3_GTP (pH to 7.3, 300 mOsm). The extracellular solution contained (in mM) 129 NaCl, 5 KCl, 30 glucose, 25 HEPES, 2 CaCl_2_, 1 MgCl_2_ (pH 7.3, 315 mOsm), and 5 µM NBQX (sigma-aldrich) was sometimes added to minimize the spontaneous seizure-like activity. In most recorded neurons, a small constant hyperpolarizing current (0–50 pA) was injected to bring the membrane potential between −75 to −70 mV.

For whole-cell patch-clamp recordings of *Drosophila* neurons, the intracellular solution contained (in mM) 140 K-gluconate, 10 HEPES, 1 KCl, 4 MgATP, 0.5 Na_3_GTP, 1 EGTA and 0.5% biocytin hydrazide (pH 7.3, 285 mOsm). The extracellular solution contained (in mM) 103 NaCl, 3 KCl, 5 *N*-tris(hydroxymethyl) methyl-2-aminoethane-sulfonic acid, 10 trehalose, 10 glucose, 2 sucrose, 26 NaHCO_3_, 1 NaH_2_PO_4_, 1.5 CaCl_2_, and 4 MgCl_2_ (pH near 7.3 when bubbled with 95% O_2_/5% CO_2_, 295 mOsm). In most recorded neurons, a small constant hyperpolarizing current (0–20 pA) was injected to bring the membrane potential between −65 to −60 mV.

For loose-patch recording of *Drosophila* projection neurons, all parameters were similar except that the electrodes (7–10 MΩ) were filled with extracellular solution, and seals of 50∼100 MΩ were formed instead of break-in. Neurons were voltage-clamped at the zero-current potential.

### Immunostaining

To visualize the recorded *Drosophila* neurons, the brains were fixed for 20 min at 25°C in 4% paraformaldehyde in PBS, rinsed with PBS, and blocked in 10% normal goat serum/PBST (0.3% Triton X-100 in PBS) for 1 hour. Brains were incubated in 1∶50 mouse nc82 antibody (DSHB) for 12 h at 4°C, and then washed in PBST for several times. After further incubation with 1∶200 goat anti-mouse Alexa Fluor 633 (Invitrogen, USA) and 1∶200 Rhodamine avidin D (Vector Laboratory, USA) for 3 h at 25°C, brains were washed for 20 min in PBST and mounted in Vectashield (Vector Laboratory, USA). Confocal fluorescence microscopy was performed on a Zeiss 5 PASCAL microscope (Carl Zeiss, Germany), using a 63× oil-immersion objective.

### Viral vector injection in vivo

Adeno-associated viral (AAV) vectors, AAV- hSynapsin-NpHR3-EYFP-2A-ChR2-mCherry, were used to drive the expression of NpHR3 and ChR2 channels specifically in cortical neurons after the in vivo injection to the brain of C57/Bl6 mouse. The AAV injection was made to the brain cortex of juvenile mice (postnatal days 14–16, P14–16), following a procedure described previously [48]. The mice were anesthetized with an intra-peritoneal injection of ketamine/medetomidine (30 and 0.36 mg/kg body weight). After the mouse was mounted on a stereotaxi (RWD Life Science), a small hole (<100 µm) in the skull was made using a dental drill (Strong) at a position 0.5 mm posterior from Bregma and 3.0 mm from the midline and the exposed dura was slightly punctured. A glass micropipette (tip size of ∼3 µm) filled with virus was then lowered to 0.7 mm below the pia surface. Using a picospritzer (Parker Instrumentation), air puffs were delivered (15 psi, 2 ms duration) at a frequency of 1 Hz to the glass micropipette to inject the virus to the cortex. The viral delivery rate was below 0.1 µl/min. The pipette was retracted 50 microns towards the surface after 50 air puffs at each depth. The injection was stopped at the depth of about 100 µm below the pia surface, and the pipette was then held in place for approximately 5 minutes before completely retracting out of the brain. The mice were recovered from anesthesia, and reared in the normal cage condition for more than a month before the experiments.

### Brain slice preparation and electrophysiology

The mouse (Thy1-ChR2-YFP transgenic line, P40–42, or mouse with virus injection, P55–60) was deeply anesthetized with sodium pentobarbital (∼100 mg/kg) and rapidly decapitated. The brain was quickly dissected and transferred into ice-cold oxygenated artificial CSF (ACSF; composed of 125 mM NaCl, 1.25 mM NaH_2_PO_4_, 2 mM CaCl_2_, 3 mM KCl, 2 mM MgSO_4_, 2.6 mM NaHCO_3_, 1.1 mM dextrose, 1.3 mM sodium ascorbate, and 0.6 mM, sodium pyruvate; pH 7.30, 300 mOSM). Coronal brain slices (330 µm thickness) were prepared with a Vibratome 3000 at 0–2 °C, and transferred to an interface holding chamber with ACSF (bubbled with 95% O2–5% CO2) for 30 min at 34 °C, followed by incubation at room temperature (25±2 °C) before use.

Whole-cell recording from neocortical neurons was made with an Axopatch 700B amplifier (Molecular Devices), using an upright microscopy (FN1, Nikon) equipped with infra-red differential interference contrast (DIC) optics. The internal solution of the recording micropipette contained (in mM) 133 K-gluconate, 9 KCl, 10 HEPES, 10 Na2HPO4, 4 MgATP, 0.3 Na_3_GTP, and 0.3 mM EGTA, adjusted pH 7.25–7.35 with KOH and to ∼300 mOSM. The pipette resistance was to 3–5 MΩ. Identifying excitatory pyramidal cells and inhibitory GABAergic fast-spiking interneurons in the neocortex was based on the firing pattern in responses to step depolarizing currents (500 ms duration) as well as the neuronal morphology under DIC. The membrane potentials or currents were recorded under the current-clamp and the voltage-clamp modes, respectively. Electric signals were filtered at 5 kHz, digitized at 10 kHz (Digidata 1440A, Molecular Devices) and acquired by a computer with pClamp 10 (Molecular Devices).

## Supporting Information

Figure S1 Illustration of the AOD-assisted laser stimulation system. The system was built based on a Nikon FN-1 upright microscope. To activate channelrhodopsin, a blue laser (∼473 nm) was introduced. A collimated laser beam sequentially passed through a half-wave plate, two crosswise-oriented AODs, and a scan lens, and then entered the optic-path of the FN-1 microscope. The laser beam was further reflected by a dichroic mirror and focused by the microscope objective to form a restricted laser spot on the focal plane (sample). Sample images were captured by a CCD camera while the sample was illuminated by high pressure mercury lamp or halogen lamp. Laser stimulation with different patterns can be achieved by the control of application software. At the same time, light-evoked responses were measured by electrophysiology recordings.
(TIF)

Figure S2 Synchronous illumination of multiple sites on neurites evoked action potentials in ChIEF-expressing cultured neurons. Pulses of laser stimulation (0.05 ms) at different intervals were provided sequentially at 12 sites on neurites adjacent (**a**) or distal (**b**) to the soma of a recorded neuron expressing ChIEF-tdTomato. Upper panels show the fluorescent images and the stimulation sites. When the interval was relatively long, only sub-threshold depolarization could be observed. However, when the interval became shorter (10 µs), the depolarizations were integrated and action potentials were evoked (red traces).
(TIF)

Figure S3 Assessment of spatial resolution of the laser activation of ChR2 to induce neuronal spike in brain slice. (A1), Color-coded plots of the mean sub-threshold depolarization amplitude and the action potential (AP) probability (measured from 10 trials) of a representative ChR2-experessing pyramidal cell (PC) in S1 slice, in response to laser pulse (0.5 ms duration) stimuli to 9 different sub-cellular domains (illustrated by the top right panel with numbers) with increasing laser power from 0.25 to 1.25 mW. Color coded value ranges: black-to-white, 0–25 mV; white-to-red: 0–100%. (A2), Averaged results of AP probability from 6 experiments shown in (A1). Stimulation locations #1–9 correspond to the numbered sub-cellular domains shown in (A1). (B1), a schematic illustrates double whole-cell recordings on two neighboring ChR2-expressing cells. Line with the end arrows: inter-neuron distance; light blue circles: laser stimulation at the soma. (B2), Superimposed AP traces from a pair of recorded ChR2-expressing PCs (with inter-neuron-distance 10 *µm*), in response to alternate laser pulse stimuli to this pair at different powers. (B3), Color-coded responses from three pairs of recorded PCs with inter-neuron-distance of 10, 20, and 30 µm. Colored scale bar is same at that shown in (A1). (C1), AP responses of ChR2-expressing PC (blue) and fast-spiking interneuron (red), respectively, in response to laser pulse stimuli (0.5 ms in duration, 0.75 mW) at 20 or 100 Hz. (C2), Averaged results from the experiments shown in (C1). Data shown in (A–B) were from brain slices of the Thy1-ChR2-YFP mice, while that of (C) were from AAV injected mice.
(TIF)
