# Supplementary figures and images for: Precise Spatiotemporal Control of Optogenetic Activation Using an Acousto-Optic Device
Source: PLoS One. 2011 Dec 9;6(12):e28468. doi: 10.1371/journal.pone.0028468 (PMC3235127; doi:10.1371/journal.pone.0028468)

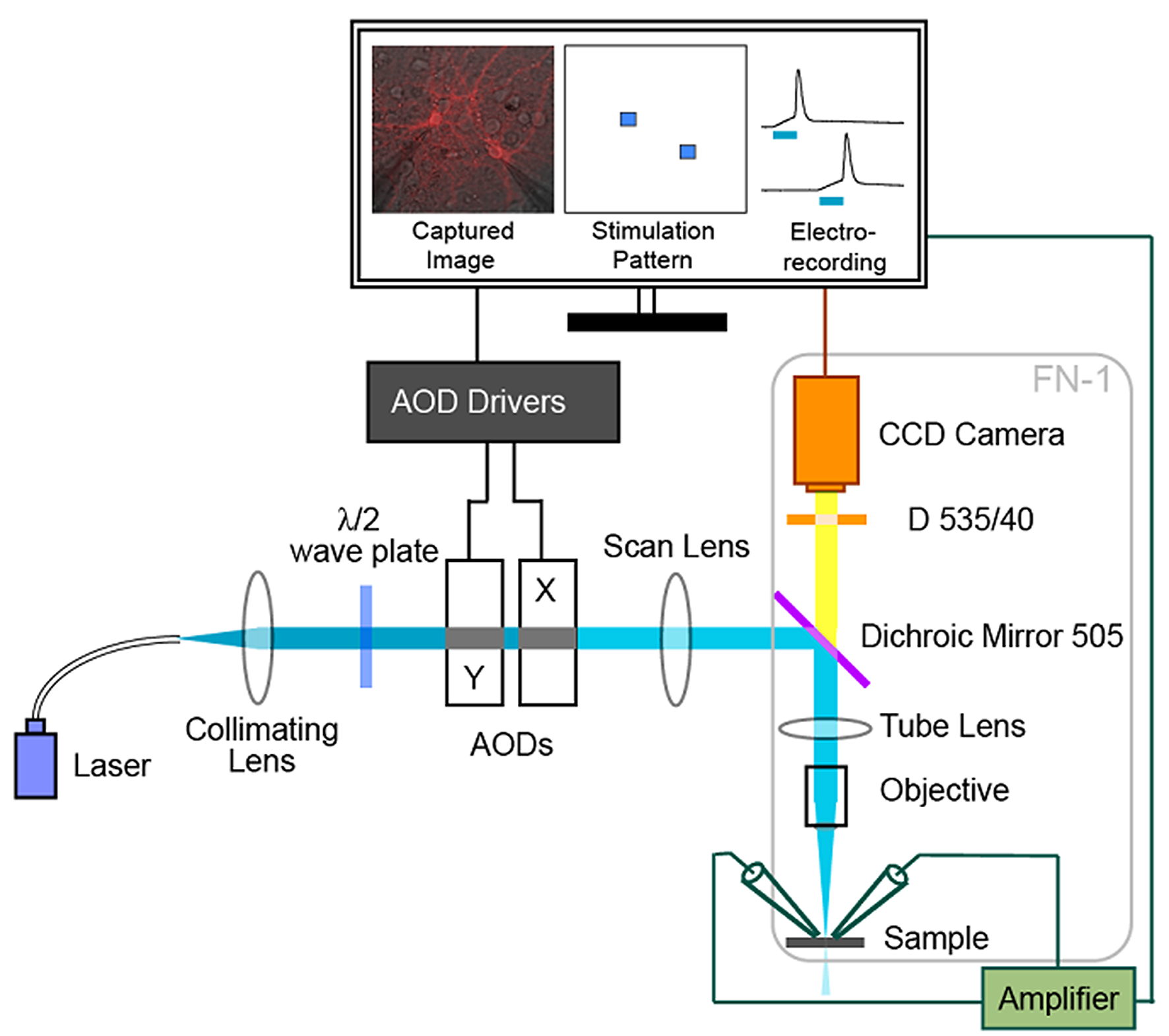

Supplement: Figure S1 — Illustration of the AOD-assisted laser stimulation system. The system was built based on a Nikon FN-1 upright microscope. To activate channelrhodopsin, a blue laser (∼473 nm) was introduced. A collimated laser beam sequentially passed through a half-wave plate, two crosswise-oriented AODs, and a scan lens, and then entered the optic-path of the FN-1 microscope. The laser beam was further reflected by a dichroic mirror and focused by the microscope objective to form a restricted laser spot on the focal plane (sample). Sample images were captured by a CCD camera while the sample was illuminated by high pressure mercury lamp or halogen lamp. Laser stimulation with different patterns can be achieved by the control of application software. At the same time, light-evoked responses were measured by electrophysiology recordings. (TIF) [file pone.0028468.s001.tif]

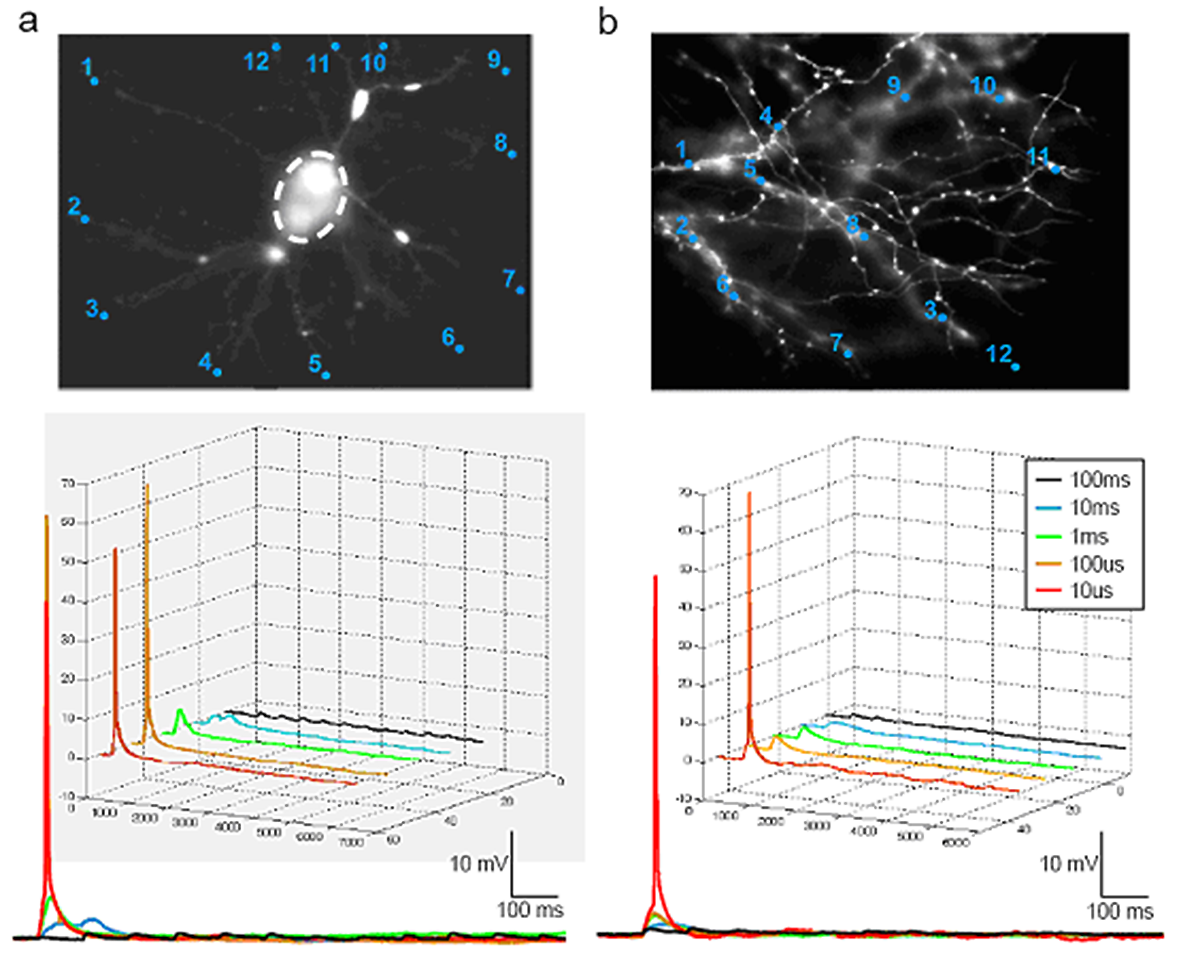

Supplement: Figure S2 — Synchronous illumination of multiple sites on neurites evoked action potentials in ChIEF-expressing cultured neurons. Pulses of laser stimulation (0.05 ms) at different intervals were provided sequentially at 12 sites on neurites adjacent (a) or distal (b) to the soma of a recorded neuron expressing ChIEF-tdTomato. Upper panels show the fluorescent images and the stimulation sites. When the interval was relatively long, only sub-threshold depolarization could be observed. However, when the interval became shorter (10 µs), the depolarizations were integrated and action potentials were evoked (red traces). (TIF) [file pone.0028468.s002.tif]

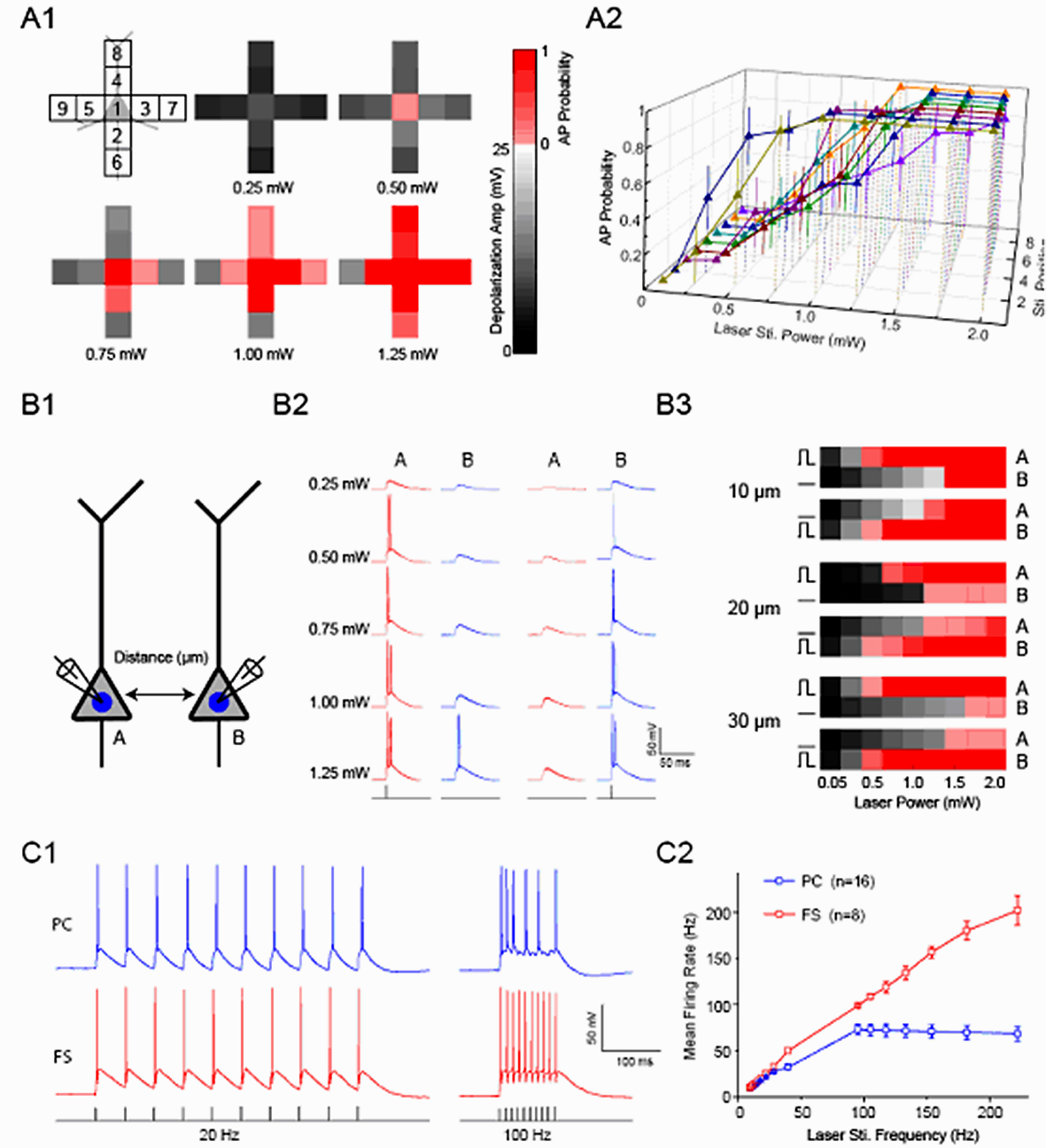

Supplement: Figure S3 — Assessment of spatial resolution of the laser activation of ChR2 to induce neuronal spike in brain slice. (A1), Color-coded plots of the mean sub-threshold depolarization amplitude and the action potential (AP) probability (measured from 10 trials) of a representative ChR2-experessing pyramidal cell (PC) in S1 slice, in response to laser pulse (0.5 ms duration) stimuli to 9 different sub-cellular domains (illustrated by the top right panel with numbers) with increasing laser power from 0.25 to 1.25 mW. Color coded value ranges: black-to-white, 0–25 mV; white-to-red: 0–100%. (A2), Averaged results of AP probability from 6 experiments shown in (A1). Stimulation locations #1–9 correspond to the numbered sub-cellular domains shown in (A1). (B1), a schematic illustrates double whole-cell recordings on two neighboring ChR2-expressing cells. Line with the end arrows: inter-neuron distance; light blue circles: laser stimulation at the soma. (B2), Superimposed AP traces from a pair of recorded ChR2-expressing PCs (with inter-neuron-distance 10 µm), in response to alternate laser pulse stimuli to this pair at different powers. (B3), Color-coded responses from three pairs of recorded PCs with inter-neuron-distance of 10, 20, and 30 µm. Colored scale bar is same at that shown in (A1). (C1), AP responses of ChR2-expressing PC (blue) and fast-spiking interneuron (red), respectively, in response to laser pulse stimuli (0.5 ms in duration, 0.75 mW) at 20 or 100 Hz. (C2), Averaged results from the experiments shown in (C1). Data shown in (A–B) were from brain slices of the Thy1-ChR2-YFP mice, while that of (C) were from AAV injected mice. (TIF) [file pone.0028468.s003.tif]
